# Supplementary material for: Bayesian robust symmetric regression for medical data with heavy-tailed errors and censoring
Source: PLoS One. 2025 Aug 1;20(8):e0329589. doi: 10.1371/journal.pone.0329589 (PMC12316244; doi:10.1371/journal.pone.0329589)
Supplement: Attachment — Submitted filename: Comments for Manuscript Number PONE-D-25-26361.docx [file pone.0329589.s001.docx]

**Major Comments**

Prior Sensitivity and Model Robustness: A critical aspect of robust Bayesian modeling is the sensitivity of the inference to prior choices. The current manuscript does not address how the selected priors, particularly those on variance components such as τ² for β and ν in the Student-t distribution, influence the results. At minimum, the authors should include a brief sensitivity analysis or alternative priors and show how these affect the posterior estimates. Alternatively, they could justify their choices using prior predictive checks or other principled arguments.

Comparative Benchmarks: The simulation study lacks comparisons to conventional or widely-used alternative methods such as Gaussian Bayesian regression, Tobit models, or even quantile regression techniques. Without such benchmarks, it is difficult to substantiate claims of superior performance. The authors should consider including these comparisons or, if not feasible, temper their conclusions accordingly.

Real Data Applications – Interpretation Deficit: The real data applications are relevant and appropriately chosen. However, the manuscript does not sufficiently interpret the results in terms of domain relevance. For example, a coefficient estimate of –0.45 for the ECOG performance status is presented without discussing what this means for patient prognosis or clinical outcomes. The authors are encouraged to provide practical interpretations that would be meaningful to clinicians or practitioners, thus increasing the utility of the findings.

**Minor Comments**

Language and Tone: In several parts of the manuscript, particularly in the Introduction, the tone is overly informal for an academic paper. Phrases like “two key things,” “in short,” and “real-world data frequently include two challenges” detract from the professional tone expected. These should be revised for consistency with academic standards.

Structure and Flow: The abstract and introduction are currently dense and could benefit from clearer paragraph structuring. This would improve readability and help highlight the main contributions more effectively. Similarly, the "Remark" under the model specification section feels disconnected from the main text and would be better integrated into the surrounding narrative.

Recent Literature: The citation list leans heavily on pre-2020 literature. Incorporating more recent developments in robust Bayesian regression, including advances in variational inference and nonparametric priors, would help situate the current work within the modern landscape of the field.

Ethical and Practical Relevance: Although the study is primarily methodological, journals such as PLOS ONE often appreciate a brief discussion on the ethical or practical relevance of the research. Since the methods are applied to medical data (e.g., survival time, hospital stay), a short paragraph highlighting the potential impact on clinical decision-making or patient care would enhance the manuscript's broader appeal.

The manuscript presents a sound methodological framework with clear relevance to the analysis of censored and noisy data, particularly in medical contexts. However, to meet the expectations of a broad-scope journal, significant revisions are needed to clarify the contribution, strengthen empirical comparisons, and improve overall presentation. I look forward to reviewing a revised and improved version.
